# Green and Sustainable Forward Osmosis Process for the Concentration of Apple Juice Using Sodium Lactate as Draw Solution

**DOI:** 10.3390/membranes14050106

**Published:** 2024-05-02

**Authors:** Yuhang Zhao, Chang Liu, Jianju Deng, Panpan Zhang, Shiyuan Feng, Yu Chen

**Affiliations:** 1 School of Environment and Resources, Southwest University of Science and Technology, Mianyang 621010, China; 2 Low Cost Wastewater Treatment Technology International Science and Technology Cooperation Base of Sichuan Province, Mianyang 621010, China

**Keywords:** forward osmosis, sodium lactate, concentration, apple juice, vacuum membrane distillation

## Abstract

China is the world’s largest producer and exporter of concentrated apple juice (CAJ). However, traditional concentration methods such as vacuum evaporation (VE) and freeze concentration cause the loss of essential nutrients and heat-sensitive components with high energy consumption. A green and effective technique is thus desired for juice concentration to improve product quality and sustainability. In this study, a hybrid forward osmosis–membrane distillation (FO–MD) process was explored for the concentration of apple juice using sodium lactate (L-NaLa) as a renewable draw solute. As a result, commercial apple juice could be concentrated up to 65 °Brix by the FO process with an average flux of 2.5 L·m^−2^·h^−1^. Most of the nutritional and volatile compounds were well retained in this process, while a significant deterioration in product quality was observed in products obtained by VE concentration. It was also found that membrane fouling in the FO concentration process was reversible, and a periodical UP water flush could remove most of the contaminants on the membrane surface to achieve a flux restoration of more than 95%. In addition, the L-NaLa draw solution could be regenerated by a vacuum membrane distillation (VMD) process with an average flux of around 7.87 L∙m^−2^∙h^−1^ for multiple reuse, which further enhanced the long-term sustainability of the hybrid process.

## 1. Introduction

China is regarded as the world’s largest apple grower and producer with a remarkable production of 47.57 million tons in 2022, accounting for more than 50% of the global market share [1]. In addition, China also leads the production and export of concentrated apple juice (CAJ) globally [2]. Typically, apple juice is extracted by pressing or squeezing from the fresh fruits to extract vitamins, minerals and other beneficial components. Meanwhile, it is necessary to perform dehydration and concentration treatment to ensure the stability of juice and to minimize the cost of the packaging, preservation and transportation of the product [3]. There are several technologies for apple juice concentration, including vacuum evaporation (VE) concentration, freeze concentration, and vacuum freeze concentration [4,5]. Among them, heat technology will cause the degradation of heat-sensitive volatile compounds and a change in the color, aroma and taste of the final product [6,7]. On the other hand, a significant loss of essential nutrients and components could happen during the removal of ice crystals in freeze concentration. Therefore, there is a growing demand for innovative technologies to replace conventional concentration methods to maintain the sensory and nutritional value of juice products, which is of great significance for the sustainable development of agriculture in China [8]. Membrane technologies such as microfiltration, ultrafiltration and reverse osmosis are widely applied in the clarification and concentration of juices without temperature and phase changes [9]. Nevertheless, it is difficult to achieve a satisfactory concentration rate with high maintenance costs on account of membrane contamination and pressure limitation. The forward osmosis (FO) process has been extensively studied for desalination [10], sewage wastewater [11,12] and irrigation [13]. It has some distinctive advantages for juice concentration such as high retention rate, low membrane fouling potential and only one-tenth energy consumption compared to the thermal process [14,15].

The draw solution (DS) is a pivotal factor in FO technology to provide a sufficient osmotic pressure gradient [16,17]. For the commonly used DS candidates, the accumulation of reverse inorganic salt deteriorates the taste of juice while the sugar solution provides nutrients for microbial reproduction in the application of juice concentration. In contrast, food additives can not only generate significant osmotic pressure but also ensure the preservation of food quality [18]. For instance, Long used a 1.5 M gluconate salt (Glu-K) for the concentration of various fruit juices through an FO process with reasonable water flux (1.6−2.6 LMH) [19]. Milczarek concentrated fresh watermelon juice to 65 °Brix using 70% glycerol without the deterioration of the desirable nutritional and sensory properties of the juice [20]. Furthermore, the regeneration of the DS is also of great importance according to the concept of cleaner production and circular economy [21,22]. Pressure-driven membrane processes demand substantial energy to overcome the osmotic pressure barrier [23,24,25]. The membrane distillation (MD) process utilizes the transmembrane vapor pressure difference to achieve clean water production and raw water concentration by hydrophobic membranes [26]. Theoretically, the water yield rate of MD can reach 100% with only the gas phase, such as water vapor passing through membrane pores, with negligible concentration effects from the raw water [27,28]. The combined FO–MD process has proven to be feasible in various wastewater treatment [29,30]. Lee conducted research on urban sewage treatment and resource recovery using MD to re-concentrate the diluted DS with a remarkable recovery rate of up to 50% [31]. Mustafa Al-Furaiji demonstrated that the FO–MD process was capable of treating extremely saline solutions containing hydrocarbons to produce high-purity water [32]. In addition, the MD process for DS regeneration would be highly cost-effective if waste heat or solar energy could be utilized [33,34].

In this study, a combined FO–MD process using sodium lactate (L-NaLa) as the DS was explored for the concentration of apple juice. The effect of different operating conditions was studied to determine the optimal parameters for the concentration process. The impacts of FO and vacuum evaporation (VE) concentration on the nutritional composition of juice were then compared. Finally, the FO membrane fouling condition was studied while vacuum membrane distillation (VMD) was applied to recover the DS to explore the long-term sustainability of the process.

## 2. Materials and Methods

### 2.1. Materials and Chemicals

FO membrane modules with an active layer at the lumen side of hollow fibers were purchased from Aromatec, Singapore. Polytetrafluoroethylene (PTFE) membranes (Zhongke Bidu New Membrane Technology Co., Ltd., Nanjing, China) with a nominal pore size of 0.1 μm were used for the VMD process. Commercially available apple juice (Huiyuan Group Co., Ltd., Beijing, China, TSS of 10.2 °Brix) was used as the feed solution (FS). Sodium chloride (NaCl) GR 99.8%, anhydrous sodium sulfite (SSF) AR 98%, sodium metabisulfite (SMB) AR 96%, sodium benzoate (SB) AR 99.5%, sodium diacetate (SDA) 99%, potassium sorbate (Sorb-K) 99% and sodium lactate (L-NaLa) (60% in water) were acquired from Macklin Biochemical Technology Co., Ltd. (Shanghai, China). to prepare the DS using ultrapure water. Gllic acid 99%, sodium carbonate anhydrous 99.9%, Folin–Ciocalteu’s phenol reagent 2 M, (+)-Catechin AR, aluminum chloride 99.99% and 2,2-diphenyl-1-picryhydrazyl (DPPH) HPLC 98.5% were also purchased from Macklin Biochemical Technology Co., Ltd. (Shanghai, China). D(+)-Glucose monohydrate was acquired from Aladdin Biochemical Technology Co., Ltd. (Shanghai, China). Sodium hydroxide AR 98%, sulfuric acid AR 95~98%, phenol GR 99.5%, sodium nitrite GR 99% and L(+)-Ascorbic acid AR 99.7% were obtained from Kelong Chemical Co., Ltd. (Chengdu, China).

### 2.2. Optimization of FO Operating Condition

**Figure 1:**
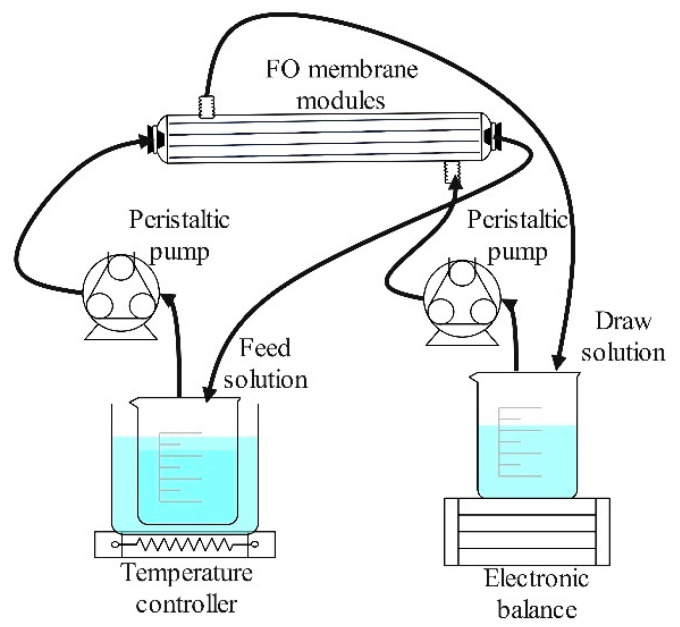
Schematic diagram of FO set-up.

The FO set-up used in this study is depicted in Figure 1. Laboratory-scale membrane modules were adopted at this stage with an effective membrane surface area of 39.56 cm^2^. The initial volumes of both FS and DS were 1 L, and the experimental duration was 1 h for each test. The flow rates of the DS and FS were adjusted by peristaltic pumps (Chuangrui Pump Co., Ltd., Baoding, China). The temperature of the FS was controlled by a thermostatic bath and a cooling water circulator (Zhixin Experimental Instrument Co., Ltd., Shanghai, China). The mass change of the DS was recorded by an electronic balance (LICHEN-BX Instrument Technology Co., Ltd., Shanghai, China). The change in the conductivity of the FS was recorded by a conductivity meter (Yidian Scientific Instrument Co., Ltd., Shanghai, China). The osmotic pressure of DS was measured using a freezing point osmometer (Advanced, Norwood, MA, USA).

UP water and 1 M NaCl solution were firstly used as the FS and DS, respectively, to evaluate the performance of the membrane module under various operating conditions, including the membrane orientation (active layer facing FS (AL-FS) mode and active layer facing DS (AL-DS mode)), FS temperature (4–35 °C), FS flow rate (25–250 mL∙min^−1^ (13.10–131.06 cm∙s^−1^)) and DS flow rate (25–250 mL∙min^−1^ (2.56–17.95 cm∙s^−1^)). Subsequently, the performance of different draw solutions including NaCl and six food additives were investigated under the optimized operating conditions using UP water as the FS. All experiments were carried out in triplicate and the average results were reported.

The water flux (L∙m^−2^∙h^−1^) was determined by the mass change of the DS as follows:

(1) $$J_{W}=\frac{m_{t,D}-m_{0,D}}{\rho A_{m}\Delta t}$$

where *m_t,D_* is the mass of the DS at time *t* in g; *m_0,D_* is the mass of the DS at time 0 in g; *ρ* is the density of water, 1.0 g∙cm^−3^; *A_m_* is the membrane effective area in m^2^; and Δ*t* is the time length in h.

The reverse solute flux (*J_S_*, g∙m^−2^∙h^−1^) refers to the net migration of the DS solute to the FS, which was calculated from the change of FS conductivity as follows:

(2) $$J_{S}=\frac{C_{t,F}V_{t,F}-C_{0,F}V_{0,F}}{A_{m}\Delta t}$$

where *C_t,F_* is the final concentration of FS in g∙L^−1^; *C*_0,*F*_ is the initial concentration of FS in g∙L^−1^; *V_t,F_* is the final volume of FS in L; *V_t,F_* is the initial volume of FS in L; *A_m_* is the membrane effective area in m^2^; and Δ*t* is the time length in h.

Specific solute flux (*J_S_/J_W_*, g∙L^−1^) is a function of the lost draw solute per unit volume of water recovered to access the potential impact of the FO process on the FS.

### 2.3. Concentration of Apple Juice

Packed apple juice with a total soluble solid (TSS) content of 10.2 °Brix was used for the concentration experiments until the TSS of the CAJ reached 65 °Brix according to the GB/T 18963-2012 standard [35]. The *concentration factor* is expressed by the content of solids in the juice as follows:

(3) $$concentration\;factor=\frac{Brix\;of\;concentrated\;juice}{Brix\;of\;the\;original\;juice}$$

In the FO concentration experiments, commercial membrane modules with an effective membrane surface area of 0.56 m^2^ were used at the recommended flow rate of 2 L∙min^−1^ (2.22 cm∙s^−1^) for DS and 2 L∙min^−1^ (10.38 cm∙s^−1^) for FS. The rest of the operating conditions were adopted as the optimal values determined earlier on, where 3 L of apple juice was used as the FS at 15 °C, and 3 L of L-NaLa was used as the DS at room temperature. Three consecutive cycles lasting approximately 6 h were carried out to explore the impact of DS replacement time and concentration. The comparative experiments of VE concentration were carried out at 65 ± 2 °C with a vacuum pressure of −0.095 MPa.

The compositional characteristics of original apple juice (OAJ) and CAJ produced by FO (CAJ-F) and VE (CAJ-V) including pH value, total acidity, nutrition and flavor compounds were analyzed. Concentrated samples were diluted back to 10.2 °Brix prior to the analysis. TSS content was determined using a hand-held digital refractometer (LICHEN-BX Instrument Technology Co., Ltd., Shanghai, China). pH measurements were made with a glass electrode/pH meter (Met-Lertoldo Instruments (Shanghai) Co., Ltd., Shanghai, China). Total acid, expressed as the weight of malic acid per liter of apple juice, was determined by a conventional titration process using 0.1 mol∙L^−1^ NaOH [36]. Total sugar was determined by the phenol-sulfuric acid colorimetric method at 490 nm with glucose as the standard [37]. Total phenolic was determined by the Folin–Ciocalteu colorimetric method at 765 nm with gallic acid solution as the standard solution [38]. Total flavonoids was determined using the aluminum trichloride colorimetric method at 510 nm with (+)-catechin as the standard [39]. The antioxidant capacity of the apple juice was determined by a 2-diphenyl-1-picrylhydrazyl (DPPH) radical scavenging assay [40]. Aroma components were analyzed by a commercial PEN 3 E-nose (Airsense, Schwerin, Germany) [41].

### 2.4. Membrane Characterization

Membrane characterizations were conducted at the inner surface of hollow fibers to assess the membrane fouling condition during the FO concentration process. Scanning electron microscopy (SEM) (Ultra 55, ZEISS, Oberkochen, Germany) equipped with an energy dispersive spectrometer (EDS) (IE450, Oxford, Oxford, England) was used to analyze the surface morphology and elemental composition of the membrane. Atomic force microscopy (AFM) (SPA300HV, Seiko, Tokyo, Japan) was used to evaluate the surface roughness of the membrane. X-ray photoelectron spectroscopy (XPS) (PHI X-tool, Ulvac-Phi, Kanagawa, Japan) was used to further identify the membrane elemental composition.

### 2.5. Recycling of Draw Solution

**Figure 2:**
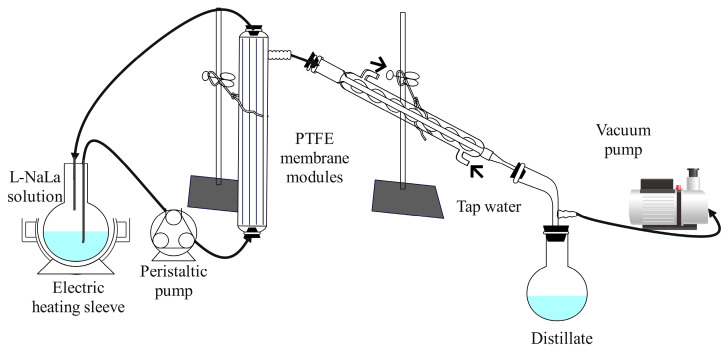
Schematic diagram of VMD set-up.

The diluted DS of L-NaLa was regenerated after the FO concentration experiments by a VMD system similar to our previous study as shown in Figure 2 [42]. PTFE membrane modules with an effective membrane surface area of 120.58 cm^2^ were used. The heat circulation system contained an electric heating sleeve (Tester Instrument Co., Ltd., Tianjin, China) and a peristaltic pump (BT300-2J, Lange Constant Flow Pump Co., Ltd., Baoding, China). The vacuum condensation system contained condensation tubes, a vacuum pump (Fujiwara Tools Co., Ltd.,Taizhou, China) and a collection bottle. The heating temperature was set at 75 ± 2 °C throughout the experiment. Membrane modules and heat circulation parts were covered with insulation cotton to reduce heat loss. Initially, the flow velocity of L-NaLa solution was set at 20 cm∙s^−1^ and reduced to 16 cm∙s^−1^ after 4 h due to the increased fluid viscosity during the concentration process. The volume and conductivity of the distillate were recorded every 30 min. The L-NaLa solution was analyzed using a UV-visible spectrophotometer (TU-1950, PERSEE, Beijing, China) to determine the compositional change during the VMD process. The L-NaLa solution was re-concentrated up to five times and re-used in the FO process to test the performance of the regenerated DS.

The permeate flux *J* (L·m^−2^·h^−1^) was used to measure the water production rate of the VMD system as follows:

(4) $$J=\frac{\Delta V}{A\Delta t}$$

where Δ*V* is the volume of produced water collected over Δ*t* in L; Δ*t* is the time interval in h; and A is the effective membrane surface area in m^2^.

The concentration factor of VMD process was measured through the volume change of L-NaLa solution as follows:

(5) $$Concentration\;factor=\frac{V_{B}}{V_{A}}$$

where *V_B_* is the volume of L-NaLa solution before VMD in L; *V_A_* is the volume of L-NaLa solution after VMD in L.

## 3. Results and Discussion

### 3.1. Effect of Operating Conditions on FO Performance

#### 3.1.1. Effect of Membrane Orientation on FO Performance

**Figure 3:**
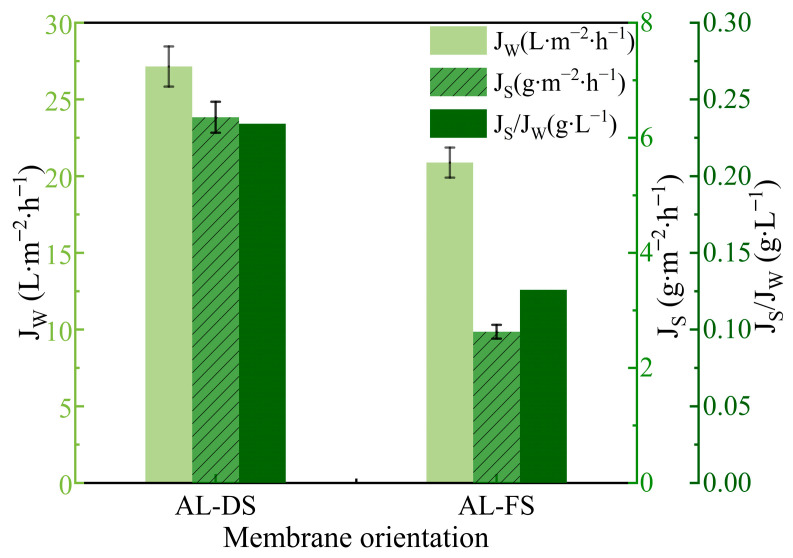
Effect of membrane orientation on FO performance. (T_FS_ = 25 °C, V_FS_ = 250 mL∙min^−1^ (131.06 cm∙s^−1^), V_DS_ = 500 mL∙min^−1^ (12.82 cm∙s^−1^)).

The FO performance under AL-DS and AL-FS modes are presented in Figure 3. A lower water flux of 21.2 L·m^−2^·h^−1^ was observed in the AL-FS mode, attributed to the occurrence of dilutive internal concentration polarization (ICP) within the support layer. The osmotic pressure difference across the membrane was therefore decreased accompanied by lower salt back diffusion [43]. On the other hand, when fruit juice was adopted as the FS in AL-DS mode, more solutes would pass through the porous structure of the membrane support layer and trapped underneath the active layer to cause more severe membrane fouling [18,44]. Therefore, the AL-FS mode is preferred in the process of juice concentration.

#### 3.1.2. Effect of FS Temperature on FO Performance

**Figure 4:**
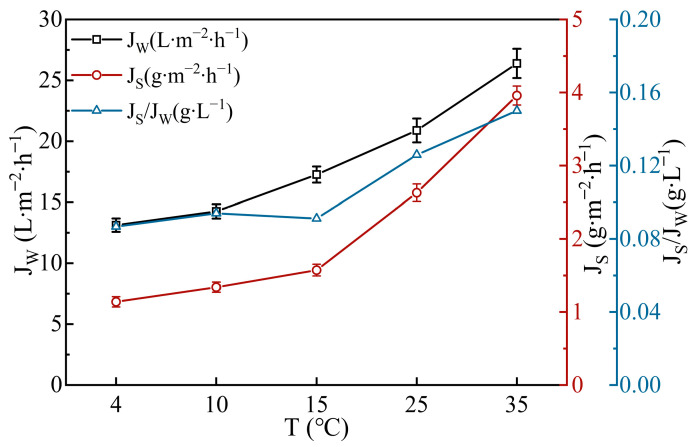
Effect of FS temperature on FO performance. (AL-FS, V_FS_ = 250 mL∙min^−1^ (131.06 cm∙s^−1^), V_DS_ = 500 mL∙min^−1^ (12.82 cm∙s^−1^)).

During the FO process, changing either the FS or DS temperature had similar effects on its performance, since heat exchange occurs between the two sides through the membrane [45]. Therefore, only the effect of FS temperature was studied, since the fruit juice required precise temperature control. Both *J_W_* and *J_S_* have an increasing trend with the rise in temperature within the experimental range in Figure 4. This was because the temperature increment led to a decrease in the viscosity of water and solute molecules resulted in higher diffusion coefficients [46]. The lowest *J_S_/J_W_* ratio was observed within the temperature range of 4–15 °C, which was considered to be the optimal condition for the FO process [47]. The FS temperature was then controlled at 15 °C for the highest *J_W_* within the range. However, it is also feasible to operate at the suitable storage temperature of 4 °C when considering long-term operation [48].

#### 3.1.3. Effect of Flow Rate on FO Performance

**Figure 5:**
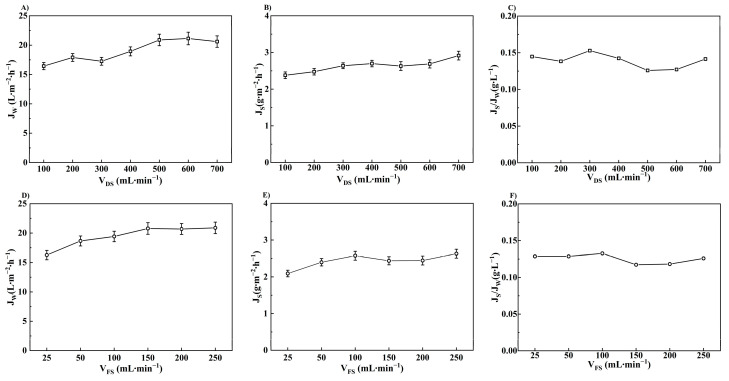
Effect of flow rate on FO performance. ((**A**–**C**): AL-FS, T_FS_ = 25 °C, V_FS_ = 250 mL∙min^−1^ (131.06 cm∙s^−1^); (**D**–**F**): AL-FS, T_FS_ = 25 °C, V_DS_ = 500 mL∙min^−1^ (12.82 cm∙s^−1^)).

The variation in FO performance at different cross flow rates is shown in Figure 5. With the increase in flow rate on both sides of the membrane, *J_W_* and *J_S_* are elevated and stabilized. *J_W_* reached its maximum value of 20.78 L·m^−2^·h^−1^ at V_DS_ = 500 mL∙min^−1^ and V_FS_ = 150 mL∙min^−1^ with the lowest *J_S_/J_W_* value of 0.12 g·L^−1^. Increasing the flow rate could enhance the renewal rate of the solution on the membrane surface to reduce the thickness of the stagnant layer. The concentration polarization was thereby mitigated, leading to an increase in the effective osmotic pressure as well as the *J_W_* [49]. However, the reverse solute flux was also enhanced at a higher flow rate due to better diffusion conditions. Therefore, a flow rate of 2 L∙min^−1^ (V_FS_ = 10.38 cm∙s^−1^ and V_DS_ = 2.22 cm∙s^−1^), as recommended by the membrane manufacturer, was used during the apple juice concentration process.

### 3.2. Effect of Draw Solution on FO Performance

DS has to be safe and harmless for human intake under the rigorous requirements of fruit juice production. Therefore, food additives are considered as the ideal candidate for the concentration process [47]. Six food additives (SSF, SMB, SB, SDA, Sorb-K, L-NaLa) were selected in this study, while their physicochemical properties are summarized in Table 1.

**Figure 6:**
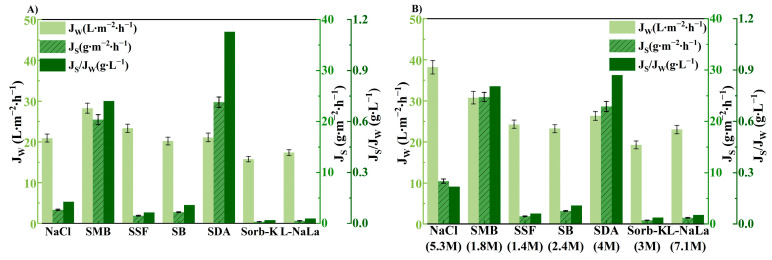
Effect of DS on FO performance (**A**) at concentration of 1 M and (**B**) at maximum concentration. (T_FS_ = 25 °C, V_FS_ = 250 mL∙min^−1^ (131.06 cm∙s^−1^), V_DS_ = 500 mL∙min^−1^ (12.82 cm∙s^−1^)).

**Figure 7:**
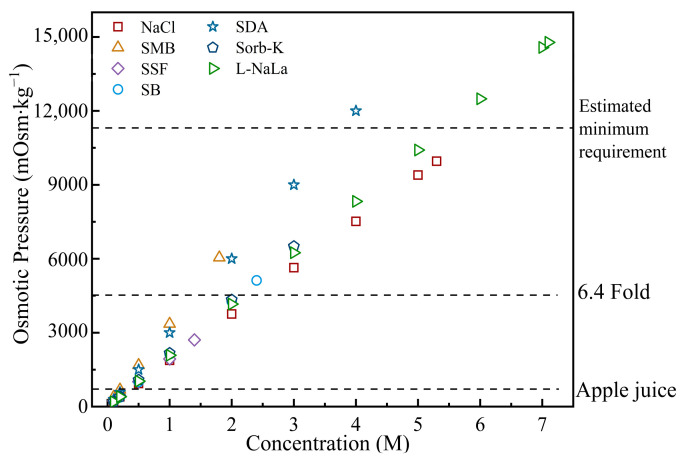
Osmolality of different draw solutions.

The FO performance using NaCl and six food additives as DSs were evaluated at the same molar concentration of 1 M at first. The water flux is in the order of SMB > SSF > NaCl > SDA > SB > L-NaLa > Sorb-K, as shown in Figure 6A. However, the *J_S_* of SDA and SMB were much higher due to the faster back-diffusion rate of H^+^ with the smallest geometrically averaged radius (1.1 Å) in comparison with other ions [51]. The saturated DSs were then tested to estimate FO performance where the maximum *J_W_* could be achieved (Figure 6B). The *J_S_/J_W_* of SSF, SB, Sorb-K, and L-NaLa were superior to those of NaCl with the *J_W_* ranging from 19.24 to 30.72 L·m^−2^·h^−1^. Furthermore, the osmolality of different DSs at various concentrations were estimated and shown in Figure 7. The osmotic pressure of all food additives except SSF were higher than the required value of 6.4 folds of CAJ. However, only approximately 30–40% of the bulk osmotic pressure difference could be utilized as the effective driving force in the FO process according to the simulation study [52]. The minimum osmotic pressure required was then estimated to be more than 11,000 mOsm∙kg^−1^. Therefore, only L-NaLa has the potential to concentrate apple juice to the target concentration with reasonable *J_W_* and excellent *J_S_/J_W_*. In addition, L-NaLa has been classified by the FAO/WHO (Joint FAO/WHO Expert Committee on Food Additives (JECFA), 1974) as a food additive that is not subject to adult upper limits [53].

**Figure 8:**
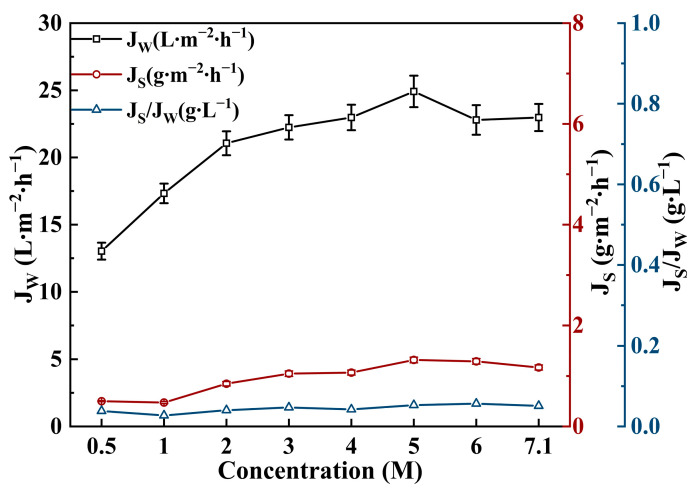
Effect of L-NaLa concentration on FO performance. (T_FS_ = 25 °C, V_FS_ = 250 mL∙min^−1^ (131.06 cm∙s^−1^), V_DS_ = 500 mL∙min^−1^ (12.82 cm∙s^−1^)).

L-NaLa was then selected for further concentration optimization study as shown in Figure 8. With the increase in L-NaLa concentration, the *J_W_* gradually increased to the maximum value at 5 M. A further concentration increment would cause the intensification of mass transfer resistance due to the higher viscosity and thus the osmotic pressure was lower than the theoretical value [47,54]. Nevertheless, the value of *J_S_/J_W_* remained at around 0.05 g∙L^−1^, even when the concentration of L-NaLa reached its saturated value. Therefore, the initial concentration of L-NaLa was set to be 5 M in the juice concentration experiments.

### 3.3. Apple Juice Concentration Experiments

#### 3.3.1. Comparative Study of FO and VE Concentration Experiments

**Figure 9:**
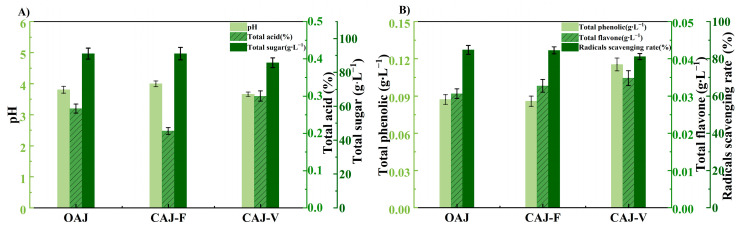
Compositional characteristics of apple juices: (**A**) pH, total acid and total sugar; (**B**) total phenolic, total flavone and DPPH radicals scavenging rate (RSR).

The compositional characteristics of pH, total acid, total sugar, total phenolic, total flavonoids, DPPH radicals scavenging rate and aroma compounds were analyzed for OAJ, CAJ-F and CAJ-V (Figure 9 and Table 2). A small amount of OH^-^ from the hydrolysis of L-NaLa was transferred to the FS during the FO process. Therefore, the pH of CAJ-F increased slightly from 3.802 to 3.994 with the total acid content decreased by 0.06%, while the rest of the measured components were similar to OAJ. In the VE method, sugar molecules underwent fragmentation or Maillard reactions when heated [55], resulting in a decrease of 5.88% in total sugar content [56]. In addition, increases in total phenolic and flavonoid contents were also observed in CAJ-V due to the inactivation of polyphenol oxidase and the cleavage of covalent bonds to the release of monomers and dimers of heat-labile compounds [57,58,59]. Furthermore, heat treatment disrupted substances such as vitamin C, catalase and anthocyanins, which providing antioxidant capacity in apple juice, to reduce the DPPH RSR value. In conclusion, the FO process had a lower impact on the juice components, resulting in better nutrient retention.

**Figure 10:**
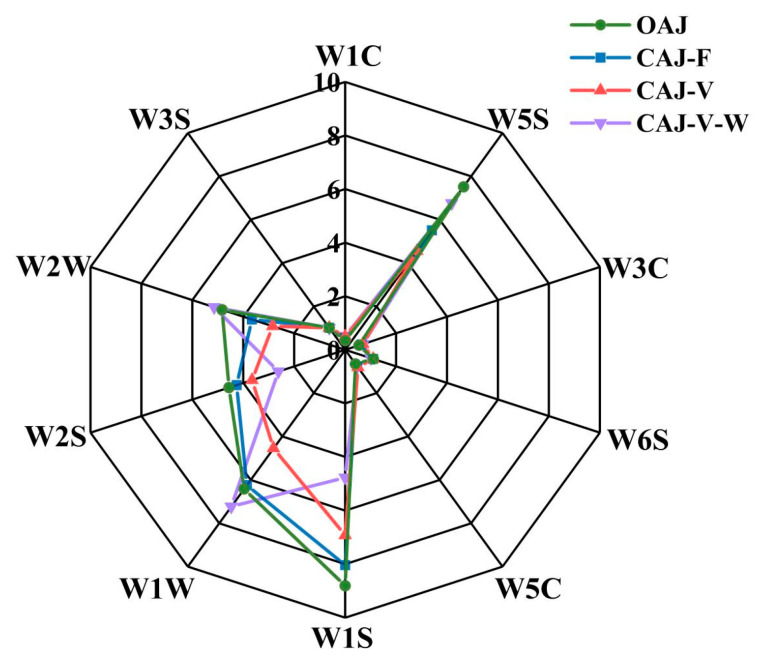
Radar chart of electronic nose response data for the apple juices.

The electronic nose is composed of 10 metal oxide semiconductor (MOS) sensors with certain selectivity for specific volatile compounds as shown in Table 3. The electronic nose radar graph (Figure 10) shows that the CAJ-F retained more aroma compounds, while the response value of CAJ-V was significantly reduced due to the decomposition and volatilization of aroma compounds in fruit juice via heat treatment [60]. To be more specific, the loss of aroma components was mainly attributed to esters and sulfur-containing (W1W, W2W) organics in apple juice [14]. The electronic nose results also indicated that FO was more capable of producing high-quality CAJ with more volatile organic compounds retained.

#### 3.3.2. Continuous FO Concentration Experiments

**Figure 11:**
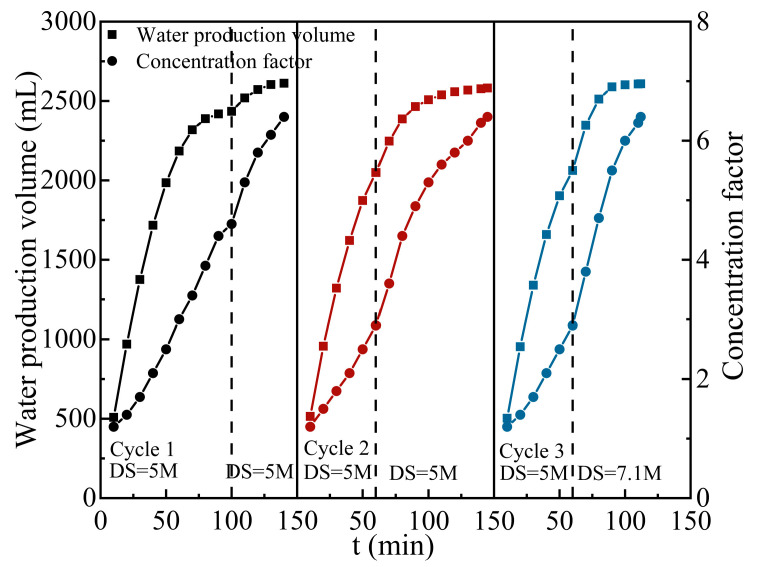
Effect of operating modes on FO performance. (T_FS_ = 15 °C; V_FS_ = 2 L·min^−1^ (10.38 cm∙s^−1^); V_DS_ = 2 L·min^−1^ (2.22 cm∙s^−1^)).

Three cycles of experiments were conducted to investigate the effect of continuous operating conditions on concentration efficiency. Each cycle was terminated when the TSS of the juice reached 65 °Brix, and the system was washed with UP water for 30 min. The dashed line in Figure 11. indicates the time point at which the DS was replaced. The water flux was significantly reduced after 100 min in the first cycle due to the dilution of the DS. Therefore, the DS was replaced with the same concentration of 5 M to reach the required 65 °Brix CAJ at 140 min. The forward shifting of draw solution replacement time to 60 min in cycle 2 showed no obvious effect in shortening the concentration time (T_2_ = 145 min), despite the fact that the process efficiency was improved for a certain period of time (60–100 min). In the third cycle, the replacement DS with a saturated concentration of 7.1 M was adopted to overcome the dilution effect at the second stage. As a result, the operating time was shortened to 112 min with an average water flux of 2.5 L·m^−2^·h^−1^, while the DS could be used for the next set of experiments with a relatively high concentration remaining (greater than 5 M). In addition, the periodic system flush by UP water was sufficient to achieve a flux restoration of more than 95% in the continuous operation.

#### 3.3.3. Membrane Autopsy Study

**Figure 12:**
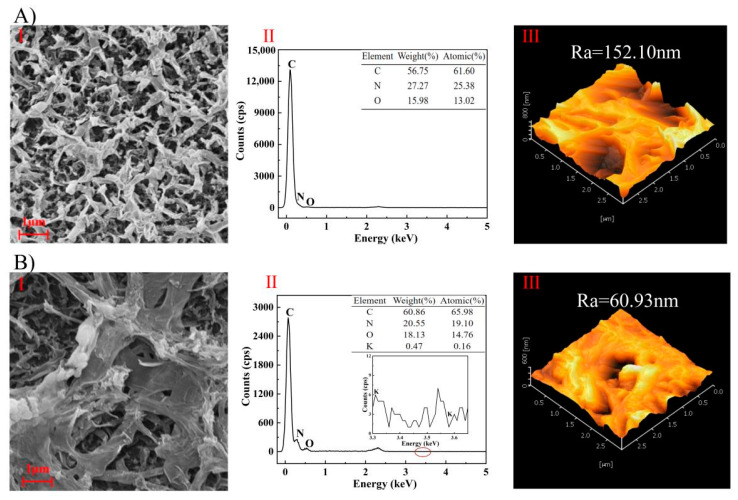
SEM-EDS and AFM results of membrane inner surface. ((**A**): original, (**B**): used; (**I**): SEM, (**II**): EDS, (**III**): AFM; Ra: average roughness).

The SEM-EDS and AFM characterization results of (A) original and (B) used membrane inner surfaces are shown in Figure 12. The inner surface of the original FO membrane had a typical RO-like structure [61]. A mud cake layer was formed on the membrane surface after the concentration experiments due to the rich organic substances in the apple juice. The increased content of C and O elements might be from the sugar ((CH_2_O)_n_) [62] and phenolic (Ar-OH) [63] components in the apple juice. The average surface roughness of the membrane surface was also significantly reduced from 152.10 nm to 60.93 nm due to the formation of the cake layer, as in Figure 12(III).

**Figure 13:**
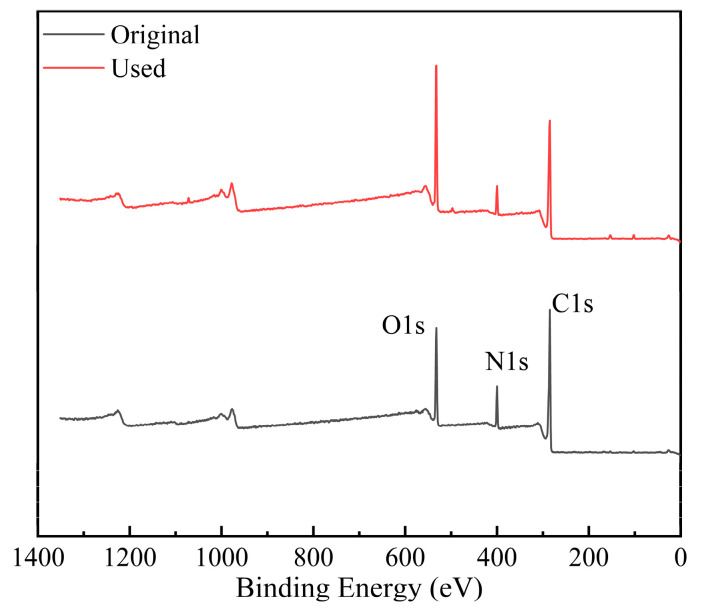
XPS full scan results of membrane inner surface.

**Figure 14:**
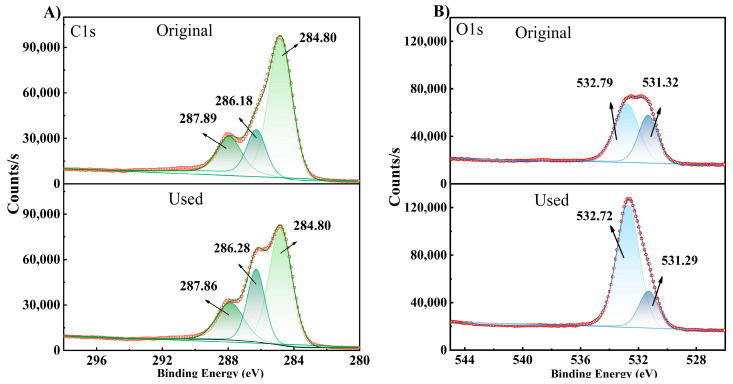
High-resolution XPS spectra of C1s (**A**) and O1s (**B**).

XPS characterization was employed to further investigate the changes in the chemical composition of the membrane surface. The full scan results were in accordance with the EDS study where the O content was increased by 10% while the ratio of C and N decreased (Figure 13 and Table 4). The high-resolution XPS spectra of C1s and O1s were further analyzed in details, as in Figure 14. The symbolic C=O (C1s: 286.2 eV/O1s:512.2 eV) from TMC, C–N (286.2 eV) from MPD and O=C–O/COOH (C1s: 287.8 eV/O1s:532.7 eV) bonds formed during the interfacial polymerization were observed on the original membrane [64,65,66]. The proportion of carbonyl carbon and carbonyl oxygen on the membrane surface were increased by 15% and 22%, respectively, after usage due to the high content of carbonyl compounds (aldehydes, ketones, reducing sugars) in the fruit juice [67].

**Figure 15:**
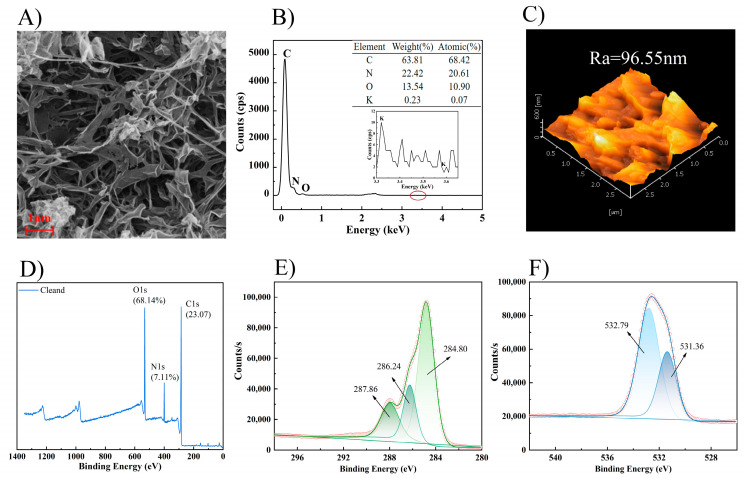
Characterization of the membrane after cleaning by UP water. ((**A**): SEM, (**B**): EDS; (**C**): AFM, (**D**): XPS full scan, (**E**): High-resolution XPS spectra of C1s; (**F**): High-resolution XPS spectra of O1s).

The characterization results of the membrane surface after UP water cleaning are shown in Figure 15. The SEM results indicated that most of the fouling layer on the membrane surface was washed off such that the distinct structure of ridges and valleys could be observed again. In addition, the rest of the characterization had all indicated that the membrane surface conditions had been restored nearly to the original state. Therefore, it could be concluded that the membrane fouling in the apple juice concentration by the FO process was mostly reversible in AL-FS mode. A periodical UP water flush would be sufficient to remove most of the contaminants on the membrane surface, as proven in the continuous operation discussed earlier on.

### 3.4. Regeneration of Draw Solution

**Figure 16:**
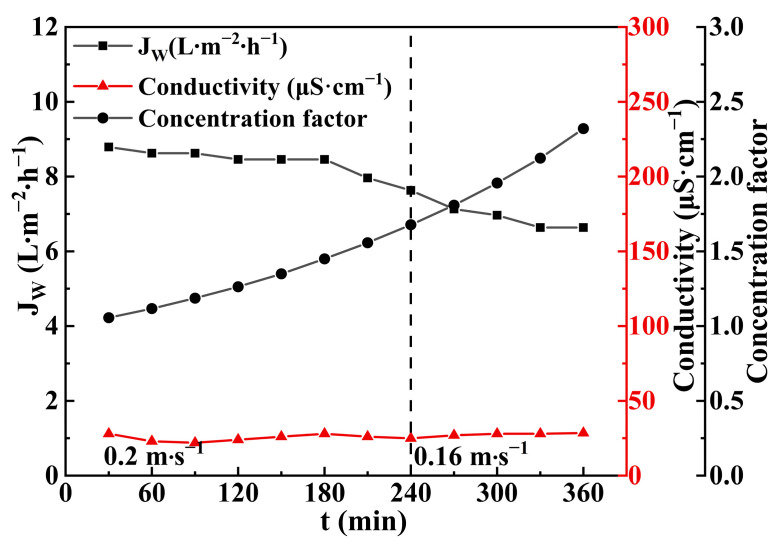
Performance of VMD regeneration of DS. (T = 75 ± 2 °C, V = 20–16 cm∙s^−1^, P = −0.095 MPa).

**Figure 17:**
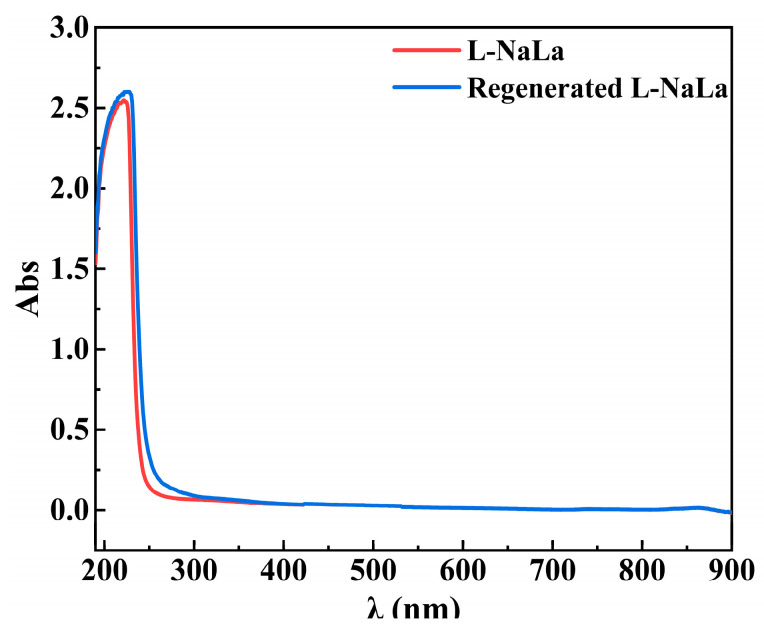
UV/Visible spectra of L-NaLa solution before and after VMD regeneration.

The performance of the VMD regeneration of DS is shown in Figure 16 (the dotted line in the figure represents the time point for adjusting the flow rate at 240 min). The DS was concentrated by a factor of 2.32 to reach the required concentration for the FO process. The water flux gradually decreased as the viscosity of the L-NaLa solution increased, with an average flux of around 7.87 L∙m^−2^∙h^−1^. Meanwhile, the low conductivity (below 30 μS∙cm^−1^) of the distillate throughout the entire process indicated that the VMD system had a high retention of L-NaLa to avoid the loss of effective components. The UV/Visible spectra in Figure 17 also showed that there was no obvious chemical degradation for the L-NaLa solution during the VMD process.

**Figure 18:**
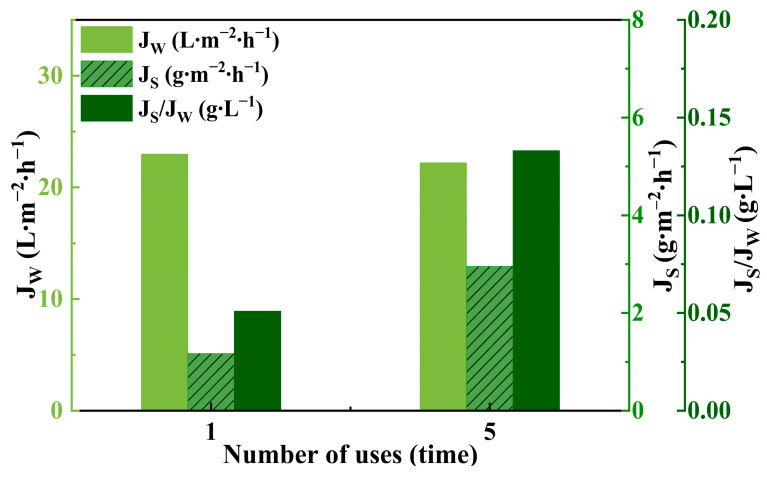
FO performance of the regenerated L-NaLa solution. (T_FS_ = 25 °C, V_FS_ = 250 mL·min^−1^ (131.06 cm∙s^−1^), V_DS_ = 500 mL·min^−1^ (12.82 cm∙s^−1^)).

The FO performance of the regenerated DS was examined by 5 cycles of FO–VMD operation with the results of first and fifth cycle shown in Figure 18 for comparison. It can be seen that the water flux of the two cycles were almost the same around 22 L·m^−2^·h^−1^. In contrast, the *J_S_* and *J_S_/J_W_* value were doubled in the fifth cycle due to the continuous release of OH^-^ from the hydrolysis of L-NaLa. However, the overall efficiency of the integrated FO–VMD process was not compromised during the continuous operation to maintain the quality of the apple juice concentrate.

## 4. Conclusions

In this study, a hybrid FO–MD process was adopted for the non-thermal concentration of commercial apple juice. A desired concentrate of 65 °Brix could be achieved by a single-stage FO under optimized operating conditions with an average flux of 2.5 L·m^−2^·h^−1^. The comparative study with VE concentration revealed that the nutrients and aroma components were well reserved in the CAJ-F compared to CAJ-V. L-NaLa, as a food additive that is not subject to adult upper limits, was also proven to be the most suitable draw solute, owing to its high osmotic pressure and low reverse salt flux. In addition, the L-NaLa draw solution could be regenerated by the VMD process with an average flux around 7.87 L∙m^−2^∙h^−1^ for multiple reuse. The membrane autopsy study showed that the membrane fouling in the continuous FO concentration process was reversible, and a periodic UP water flush could remove most of the contaminants to achieve a flux restoration of more than 95%. This work suggested that the hybrid FO–MD process has great potential in the practical application of juice concentration for improved product quality and process sustainability.

## Figures and Tables

**Table 1:** Physicochemical properties of food additives.

Additive	M_W_ (Da)	pH (1 M, 25.0 ± 0.5 °C)	Max Solubility in Water [50] (M)	Estimated Osmotic Pressure at Saturation Concentration ^a^ (mOsm∙kg^−1^)	Safety Limit ^b^ (g∙kg^−1^)
Sodium chloride (NaCl)	58.4	6.5	5.3	9959	N.A.
Sodium metabisulfite (SMB)	190.0	4.5	1.8	6044	0.07
Sodium sulfite (SSF)	126.0	10.5	1.4	2708	0.07
Sodium benzoate (SB)	144.1	9.8	2.4	5122	0.20
Sodium diacetate (SDA)	142.0	5.0	4.0	12,000	0.50
potassium sorbate (Sorb-K)	150.2	10	3.0	6504	1.00
Sodium lactate (L-NaLa)	112.1	7.2	7.1	14,782	N.A.

^a^. Estimated by the osmotic pressure at 0.5 M multiply by its max solubility in water due the detection limits of the instrument. ^b^. Food Additives Codex General Standard of the World Food and Agriculture Organization.

**Table 2:** Compositional characteristics of apple juices.

Element	OAJ	CAJ-F	CAJ-V	Benefits
pH	3.802	3.994	3.658	a. Color stability b. Stability of antioxidant substances c. Taste and flavor
Total acid (%)	0.266	0.206	0.300	a. Nutritive value b. Food preservation
Total sugar (g∙L^−1^)	91.03	91.03	85.67	Energy supply
Total phenolic (g∙L^−1^)	0.087	0.086	0.115	a. Flavor and aroma b. Antibacterial and anti-inflammatory
Total flavone (g∙L^−1^)	0.031	0.033	0.035	a. Antioxidant b. Lowering blood lipids and blood pressure
DPPH RSR (%)	84.78	84.47	81.13	Antioxidant

**Table 3:** Sensors used and their main application in PEN3.

Sensor Name	Type of Substance
W1C	Aromatic
W5S	Broad range
W3C	Aromatic
W6S	Hydrogen
W5C	Arom–aliph
W1S	Broad methane
W1W	Sulphur–organic
W2S	Broad alcohol
W2W	Sulph–chlor
W3S	Methane–aliph

**Table 4:** Elemental composition of membrane inner surface.

Sample	C (Atomic%)	N (Atomic%)	O (Atomic%)
Original	69.81	10.23	19.96
Used	64.29	7.11	28.6

## Data Availability

The data in this study are available on request from the corresponding author.

## Abbreviations

CAJ	concentrated apple juice
VE	vacuum evaporation
FO	forward osmosis
MD	membrane distillation
L-NaLa	sodium lactate
UP	ultrapure
DS	draw solution
Glu-K	Potassium gluconate
VMD	vacuum membrane distillation
PTFE	polytetrafluoroethylene
FS	feed solution
NaCl	sodium chloride
SSF	sodium sulfite
SMB	sodium metabisulfite
SB	sodium benzoate
SDA	sodium diacetate
Sorb-K	potassium sorbate
DPPH	2,2-diphenyl-1-picryhydrazyl
SEM	scanning electron microscope
EDS	energy dispersive spectrometer
AFM	atomic force microscopy
XPS	X-ray photoelectron spectroscopy
TSS	total soluble solid
OAJ	original apple juice
CAJ-F	CAJ produced by FO
CAJ-V	CAJ produced by VE
ICP	internal concentration polarization
RSR	radicals scavenging rate
MOS	metal oxide semiconductor
CAJ-V-W	distillate produced in VE
TMC	trimesoyl chloride
MPD	m-Phenylenediamine
